# Notes and News

**Published:** 1900-01-13

**Authors:** 


### Notes and News.

Mr. Thomas King, of Tisbury, Wilts, has bequeathed £1,000 to the Metropolitan Hospital Sunday Fund.

Efforts are being made by medical men in Berlin to secure the admission of women doctors as members of the Berlin Medical Society.

Mr. Armistead is a generous friend to the Dundee Royal Infirmary. In little more than a year he has given £2,500 to the institution.

Influenza is very widely prevalent at the present time in England, and those who try to escape it will find that one favourite resort, namely, the Riviera, has been invaded by it also.

Surgeon-General Jameson, C.B., has become the recipient of a medal from the Emperor of Germany, founded by him in memory of his grandfather, the Emperor William. Surgeon-General Jameson was in charge of the English ambulance in the Franco-Prussian war.

Glasgow is very desirous of rendering important assistance to the sick and wounded in the war. The provision of a hospital ship has been proposed. and a large corps of medical volunteers have offered their services. The men mostly hold first aid certificates, and have spent several seasons training with the regular forces at Aldershot and Netley Hospital.

Improvements and additions have just been completed at the Redhill and Reigate Cottage Hospital. The accommodation has now been increased to 30 beds. The new west ward is a replica of that situated on the east side of the building. The whole cost of the new portion of the hospital and its furnishing, the restoration of the roof, and other improvements, has been £1,600.

The proprietors of The Hospital have had much pleasure in offering copies of "First Aid to the Injured and Management of the Sick," by E. J. Lawless, M.D., and published by the Scientific Press, to the contingent of the London Rifle Brigade now proceeding to the war. In response, Major Tod writes for Colonel Cholmondeley that "he would be extremely grateful if you would consign 200 copies to him on board the steamer 'Briton' at Southampton." The directions given in the book are clear, and couched in simple language, and will probably be found very useful, as a soldier may be called at any time to render first aid to a comrade on the field, and when his efforts are directed by even elementary knowledge they will be doubly valuable.

The late Mr. Manasseh Gledhill has left about £15,000 to institutions in Lancashire, £12,500 of which goes to Lancashire hospitals and homes.

The Queen's Hospital, Birmingham, has received £5,000, the result of the Birmingham Hospital Sunday Fund collection. The collection of the fund during the past year was the largest for 21 years past.

The steamer "J. W. Taylor," among the crew of which there were several cases of bubonic plague while she was on her voyage from Santos to New York, arrived recently at Queenstown from the latter port. No sickness occurred during the voyage from New York.

At the last quarterly meeting of the Metropolitan Hospital Saturday Fund the report of the Finance Committee showed that the receipts from the workshops and business houses in London had amounted to £15,176 in 1899, as compared with £14,812 collected in 1898.

The plague has now nearly disappeared from Portugal. In Bombay there are weekly a large number of cases, and the returns show no steady diminution. The reports in other quarters are better, but it is to be hoped that the threatening famine will not lend its assistance to the disease.

Sir John Sibbald has been presented with a portrait of himself by a number of his friends. The painting is by Sir George Reid, president of the Royal Scottish Academy. The Chairman of the Board of Lunacy presided on the occasion of the presentation, and a number of distinguished medical men were present.

Telegrams from Melbourne, dated December 28th, state that it is announced from Numea that five Kanakas had died there from plague, and that eigh others had been placed in quarantine, while four whites who had become infected were progressing favourably.

The committee of the hospital ship "Maine" installed a complete set of the Dowsing radiant heat apparatus for the treatment of soldiers suffering from rheumatism and kindred ailments. Two male nurses were sent to Messrs. Dowsing's establishment at 7, Lower Seymour Street, and instructed in the use of the various appliances. The American doctors were highly pleased with the apparatus, and expressed their opinion that it would be of great service to them for general medical purposes on board ship where the application of heat was essential, especially as the usual spirit-lamp is generally objected to on board.

There has been a falling off in the Manchester Hospital Sunday and Saturday Fund collections this year. When addressing the annual meeting we are glad to see that those who addressed the gathering did not attribute the decrease to the existence of the war funds, a plea that is so hastily put forward at the present time. Another gentleman suggested that the fund should supply the clergy with information and fresh material to aid them in their appeals. This proposal coincides with our own practice, which is to collect striking figures and facts in our Hospital Sunday number, and they have proved very useful to many busy ministers of religion in the metropolis, many of whom have not the means of securing them for themselves.

